# The sustainable development goals, violence and women’s and children’s health

**DOI:** 10.2471/BLT.16.172205

**Published:** 2016-05-02

**Authors:** Claudia García-Moreno, Avni Amin

**Affiliations:** a World Health Organization, avenue Appia 20, 1211 Geneva 27, Switzerland.

The sustainable development goals (SDGs), which are defined in the 2030 Agenda for Sustainable Development,^1^ offer an opportunity to achieve the commitments on the prevention of violence against women made by governments in many previous declarations, resolutions and international agreements.

One of the goals related to violence is SDG 5, which addresses gender equality and the empowerment of women and girls. This goal includes two specific targets on elimination of violence and harmful practices. Target 5.2 is set out to “eliminate all forms of violence against all women and girls in public and private spheres, including trafficking and sexual and other types of exploitation”. The goal of target 5.3 is to “eliminate all harmful practices, such as child, early and forced marriage and female genital mutilation”. The inclusion of these specific targets is a recognition that addressing all forms of violence and harmful practices against women and girls is central to achieving gender equality and women’s empowerment, which is essential for sustainable development. In addition, another goal, SDG 16, which promotes peace, justice and strong institutions for sustainable development, calls in target 16.2, for an end to abuse, exploitation, trafficking and all forms of violence against children. This is the first time that a global development agenda has addressed all forms of violence against women and girls, as well as violence against children.

The social and economic costs and the negative impact of violence on women’s and children’s health and well-being are well documented.^2^^,^^3^ Women who are experiencing violence or at risk of violence need to have access to quality services, including medical care and psychosocial support, housing, police and legal services. Evidence suggests that women who are experiencing or have experienced violence make higher use of health-care services.^4^ Health systems and health-care providers therefore play an important role in providing survivors of violence with support, care and treatment, including for injuries and for sexual, reproductive and mental or any other health problems. Most women at some point in their lives seek reproductive health care, for example for contraception, post-abortion care and maternal or child health care including antenatal care, delivery or postnatal care. As such, reproductive health services in particular offer opportunities for identification and provision of a supportive response for women who have experienced violence.

An empathetic, supportive response can facilitate women taking action to address the violence they are experiencing and start healing.^5^ Health services can also facilitate women’s access to other services including social services such as housing, legal or police services. However, in many countries the health sector remains less engaged in the multisectoral response to prevent and respond to violence, compared to other sectors. Advocacy and political will is required to ensure that the health sector plays its role in addressing violence against women.

In May 2014, the World Health Assembly adopted a resolution titled: “Strengthening the role of the health system in addressing violence, in particular against women and girls and against children.”^6^ The resolution asks the World Health Organization (WHO) to develop a global plan of action on strengthening the role of the health system in addressing interpersonal violence, in particular against women and girls and against children. Over the last year, WHO has developed the action plan through extensive consultations with ministries of health, civil society, UN agencies and other relevant stakeholders. In January 2016, the WHO Executive Board approved the action plan for adoption at the World Health Assembly in May 2016.^7^ The action plan provides an important platform for strengthening and scaling up the contribution of public health towards the achievement of SDGs 5.2, 5.3 and 16.2.The action plan aims to guide Member States, other partners and WHO on actions needed to prevent violence against women and against children and to provide comprehensive quality health services and address the negative health consequences.^7^ It also covers actions for strengthening the health system’s political commitment, as well as its role in data collection and strengthening evidence to inform actions.

Implementation of the action plan needs to be guided by principles of gender equality, human rights and health equity. This will require health leaders and policy-makers to publicly challenge the social acceptability of violence against women and against children. Governments will need to integrate violence prevention and response programming into national health plans and strategies and allocate human and financial resources for their implementation. Governments will also need to provide people-centred health services to survivors, strengthen partnerships and coordination for a multisectoral response that enables communities to participate.

In 2013, WHO issued clinical and policy guidelines for responding to intimate partner violence and sexual violence against women.^8^ The guidelines provide recommendations on identification and clinical care for survivors of intimate partner violence, clinical care for survivors of sexual violence and guidance on how to organize services and training for health-care providers. Governments can use these guidelines as the basis for developing or updating their national guidelines and protocols for health sector response to violence against women. National guidelines and protocols need to be accompanied by provision of accessible, affordable, quality and comprehensive health services for women and children, including those living in humanitarian settings. These health services need to include first-line support (based on psychological first aid), care for injuries, mental health, sexual and reproductive health or any other health problems. For women who have been raped, services need to include emergency contraception, safe abortion to the full extent of the law, prophylaxis for sexually transmitted infections and human immunodeficiency virus, hepatitis B vaccination and mental health care. Strengthening the capacity of health-care providers to respond effectively to those affected by violence is central to quality of care. Integrating the prevention of and response to violence against women in medical, nursing and midwifery curriculum, as well as in in-service trainings is critical to improving service provision. Health-care providers need skills in several areas, for example, how to inquire about violence in a sensitive way and how to provide appropriate care. It is also important to address health-care providers’ attitudes towards violence and towards survivors.

Another priority in the action plan is prevention of violence. Multisectoral programming to promote gender equality and women’s empowerment is central to the prevention of violence against women and girls. The health sector can support programmes that challenge social norms and attitudes that condone male violence against women and that perpetuate male power and dominance over women, children or other men.^9^ It can also advocate with other sectors to implement programmes and policies to improve access to quality secondary education for girls, safe employment or other strategies for women to generate and own their income. 

Evidence shows that children exposed to violence are more likely to perpetuate violence later in life and also to have behavioural, emotional and mental health problems during their childhood and adolescence.^3^^,^^9^ Therefore, addressing violence against children is important for child health and development and also an important approach to preventing violence against women. Interventions for preventing child maltreatment, such as parenting programmes and home visitation programmes targeting families-at-risk have shown promise.^3^ The health sector can contribute to preventing violence against women by providing psychosocial support to children that are exposed to their mother being abused. This requires strengthening mental health services more broadly and increasing the availability of low-cost psychotherapeutic or psychosocial interventions for the mother and the child or both.^3^ The health sector can also contribute to violence prevention by addressing risk factors such as alcohol and substance abuse.

Implementing the action plan also requires robust information and evidence as the basis for developing programmes and policies. Here, it is vital that governments strengthen routine reporting on violence against women across all ages and monitor progress in implementing the health sector’s response. Specific indicators on violence against women should be included in health information and surveillance systems. To monitor the progress in achieving SDG 5.2, one proposed indicator is prevalence of intimate partner violence. Governments will need to establish baseline data for this and other indicators, and invest in systematic collection of data on violence against women through conducting population-based surveys.

There has been steady, albeit slow, progress in accumulating evidence of which strategies work, or are promising, to prevent and respond to violence against women.^10^ However, the evidence needs to be strengthened considerably and hence, investments are needed in research to develop, pilot, evaluate and scale up interventions for both prevention and response. Accountability for implementing the action plan rests with governments. Progress in implementation can be monitored, for example, by tracking the number of countries that include prevention programmes and services for violence against women in their national health plans or policies. Other indicators to monitor progress could be the coverage of services for sexual violence survivors and the number of countries that have a nationally representative survey on prevalence of violence against women.

We need to foster safe, nurturing and equitable relationships for all, which consequently will improve health, ensure a safer and fairer world for all and thereby promote sustainable development. Reductions in violence against women and violence against children will also contribute to achieving many of the other SDGs targets.
